# Neonatally Induced Mild Diabetes in Rats and Its Effect on Maternal, Placental, and Fetal Parameters

**DOI:** 10.1155/2012/108163

**Published:** 2012-06-20

**Authors:** Yuri Karen Sinzato, Gustavo Tadeu Volpato, Isabela Lovizutto Iessi, Aline Bueno, Iracema de Mattos Paranhos Calderon, Marilza Vieira Cunha Rudge, Débora Cristina Damasceno

**Affiliations:** ^1^Laboratory of Experimental Research in Gynecology and Obstetrics, Department of Gynecology and Obstetrics, Botucatu Medical School, Universidade Estadual Paulista (Unesp), Distrito Rubião Júnior 18618000 s/n, Botucatu, SP, Brazil; ^2^Institute of Biological and Health Sciences, University Center of Araguaia, Mato Grosso Federal University (UFMT), Rodovia BR-070, Km 5, 78600-000 Barra do Garças, MG, Brazil

## Abstract

The aim of this study was to assess placental changes and reproductive outcomes in neonatally induced mild diabetic dams and fetal development in their offspring. At birth, female rats were assigned either to control or diabetic group (100 mg of streptozotocin/Kg, subcutaneously). At adulthood, the female rats were mated. During pregnancy, the blood glucose levels and glucose and insulin tolerance tests were performed. At term, maternal reproductive outcomes, fetal and placental weight, and placental morphology were analyzed. Diabetic rats had smaller number of living fetuses, implantations and corpora lutea, and increased rate of embryonic loss. Placenta showed morphometric alterations in decidua area. Our results showed that mild diabetes was sufficient to trigger alterations in maternal organism leading to impaired decidua development contributing to failure in embryonic implantation and early embryonic losses. Regardless placental decidua alteration, the labyrinth, which is responsible for the maternal-fetal exchanges, showed no morphometric changes contributing to an appropriate fetal development, which was able to maintain normal fetal weight at term in mild diabetic rats. Thus, this experimental model of diabetes induction at the day of birth was more effective to reproduce the reproductive alterations of diabetic women.

## 1. Background

Pregnant women with type 1 or type 2 diabetes are at increased risk of miscarriage, stillbirth, congenital malformations, placental abnormalities, and intrauterine malprogramming. Despite current treatments, maternal diabetes is an unfavorable environment for embryonic and fetoplacental development [1–7]. These important aspects of human diabetic pregnancies can be studied using the appropriate animal models [8], not only by ethical reasons but also by the multiplicity of uncontrolled variables that may modify the intrauterine environment [9]. 

Experimental models of severe diabetes (glycemia > 300 mg/dL), which reproduce the clinical conditions of poorly controlled type-1 diabetes, have been widely used [10–14]. However, only a few studies have evaluated the repercussions of diabetes on pregnant rats and/or their offspring [9, 15–18] using models of mild diabetes (glycemia between 120 and 300 mg/dL). In a previous study conducted at our laboratory [19], experimental mild diabetes induced at 5 days of life was not effective in reproducing the reproductive outcomes (miscarriage, fetal viability, and morbidity) observed in diabetic pregnant women. Although glycemic levels were consistent with those reported elsewhere, a large number of animals were not able to mate while others did not reach at term pregnancy. In addition, in the cases that rats reached at term pregnancy, no repercussions were observed probably due to a maternal adaptive response to mild glycemic levels or placental changes. Thus, we hypothesized that another model of mild hyperglycemia (diabetes induced at day 0 of birth) could better reproduce the repercussions in the maternal reproductive parameters and fetoplacental development. Therefore, the aim of this study was to assess placental changes and reproductive outcomes in neonatally induced mild diabetic dams and fetal development in their offspring.

## 2. Methods

### 2.1. Animals and Experimental Groups

Procedures and animal handling were performed in accordance with the guidelines provided by the NIH Guide for the Care and Use of Laboratory Animals and authorized by the Ethics Committee for Animal Research of the Universidade Estadual Paulista (UNESP) São Paulo.

Wistar rats obtained from the UNESP Vivarium (São Paulo, Brazil) were used. The rats were maintained in an experimental room under controlled conditions of temperature (22 ± 2°C), humidity (50 ± 10%), and a 12-hour light/dark cycle with *ad libitum* access to commercial diet (Purina rat chow) and tap water. Parental female rats were mated to obtain newborns (NBs). On the day of birth, the rats were randomly distributed into two experimental groups: nondiabetic (control) and mild diabetic rats (MD). 

### 2.2. STZ Administration

Mild diabetes was induced at birth by subcutaneous administration of streptozotocin (STZ—SIGMA *Chemical Company, *St. Louis, MO), at a dose of 100 mg/kg of body weight diluted in 0.1 mol/L of citrate buffer (pH 4.5) [17, 20]. The rats in the control group received only citrate buffer. Following induction, the rats were maintained with their mothers (a maximum of eight females) during the lactation period (21 days). At the end of this period, the mothers were killed by carbon dioxide inhalation, and the offspring were maintained in the Laboratory of Experimental Research in Gynecology and Obstetrics under controlled conditions.

### 2.3. Mating Period

At adult age (110 days of life), the female rats were mated with nondiabetic males. The morning when spermatozoa were found in the vaginal smear was designated gestational day 0. 

### 2.4. Pregnancy Period

#### 2.4.1. Glycemia

For inclusion in this study, rats from the MD group presented glycemia between 120 and 300 mg/dL (mild diabetes) [18, 21] and rats from the control group glycemia <120 mg/dL at day 0 of pregnancy. During pregnancy, the females were maintained in individual cages. In the mornings of days 0, 7, 14, and 21, blood glucose levels (food *ad libitum* overnight) were monitored using a specific glucose meter (OneTouch Ultra—Johnson & Johnson), and the values were expressed in milligrams per deciliter (mg/dL).

#### 2.4.2. Insulin Tolerance Test (ITT)

At day 15 of pregnancy, to catabolic phase of rat pregnancy, after a 6-hour fasting period, a subcutaneous insulin tolerance test was performed. ITT consisted of a bolus injection of insulin solution (3.33 U/mL) in the dorsal area (30 *μ*g/100 g body weight). Blood samples were obtained from a cut tip tail for serum glucose determinations using a specific glucose meter (OneTouch Ultra—Johnson & Johnson) at 0, 30, 60, and 120 min [22, 23]. 

#### 2.4.3. Oral Glucose Tolerance Test (OGTT)

An oral glucose tolerance test was performed at day 17 of pregnancy still to catabolic phase of rat pregnancy. After fasting for 6 hours, a glucose solution (200 g/L) was administered by *gavage* at a final dose of 2 g/kg body weight. Blood samples were obtained from a cut tip tail for glycemic determinations using a specific glucose meter (OneTouch Ultra—Johnson & Johnson) at 0, 30, 60, and 120 min [22, 23]. Glucose responses during the glucose tolerance test were evaluated by estimation of the total area under the curve (AUC), using the trapezoidal method [24].

#### 2.4.4. Maternal Reproductive Performance

At day 21 of pregnancy, the dams were weighed and lethally anesthetized with sodium thiopental (Thiopentax). The laparotomy procedure was performed with exposure of the uterine horns. The uterus was removed and weighed to determine the litter weight, and the ovaries and uterine contents were examined to determine the number of corpora lutea, implantation, resorptions (embryonic death), and number of viable or dead fetuses. The preimplantation loss rate was calculated by the following formula: number of corpora lutea−number of implantations × 100/number of corpora lutea. The postimplantation loss rate was calculated by the following formula: number of implantations−number of living fetuses × 100/number of implantations [25]. Fetuses at term were removed and weighed. 

#### 2.4.5. Placental Morphometry and Histopathological Analyses

After weighing, the placentas were sectioned medial sagittally and fixed in 10% buffered formalin before being processed for paraffin embedding. Formalin-fixed placentas were dehydrated in a graded ethanol series and embedded in paraffin according to a standard protocol. One placenta of each dam was chosen randomly for the morphometry and histopathological analyses. The placental blocks were sectioned at longitudinal sections and mounted on glass slides for hematoxylin-eosin staining. From each slide, six fields per placenta were examined, avoiding the areas of infarction and histological artifacts. The morphometric analysis was performed on a computerized system with photomicroscope-coupled imaging (DM 2500, Leica) using Leica QWIN software, version 3.3.0. The mean areas of each placental zone were evaluated in square millimeters (mm^2^). The slides were examined at 25x magnification for measuring the labyrinth area and 100x magnification for measuring the areas of the junctional and decidual zone. The histopathological analyses considered parameters as inflammatory infiltrations, cystic spaces with transudate, hemorrhagic areas, and number of glycogen cells. 

### 2.5. Statistical Analysis

The results were presented as mean standard deviation (SD), and the comparisons between groups were performed using One-way ANOVA followed by the Student *t*-test. The chi-square test was used for comparison of proportions. Statistical significance was considered as *P* < 0.05.

## 3. Results

### 3.1. Blood Glucose Levels, Oral Glucose Tolerance Test, and Insulin Tolerance Test

**Figure 1:**
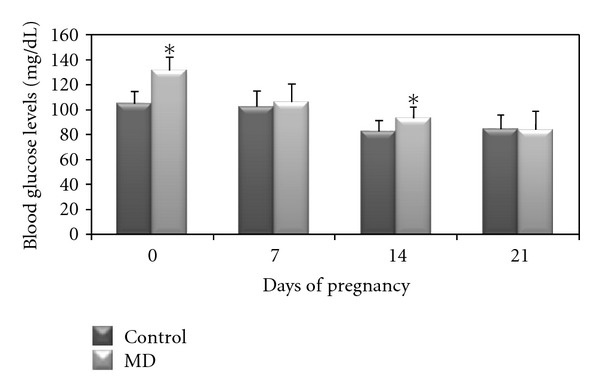
Glycemic evaluation of rats with mild diabetes (MD) and nondiabetic rats (Control). Data expressed as mean standard deviation. *P < 0.05—statistically significant difference compared to control group (Student *t*-test).

**Figure 2:**
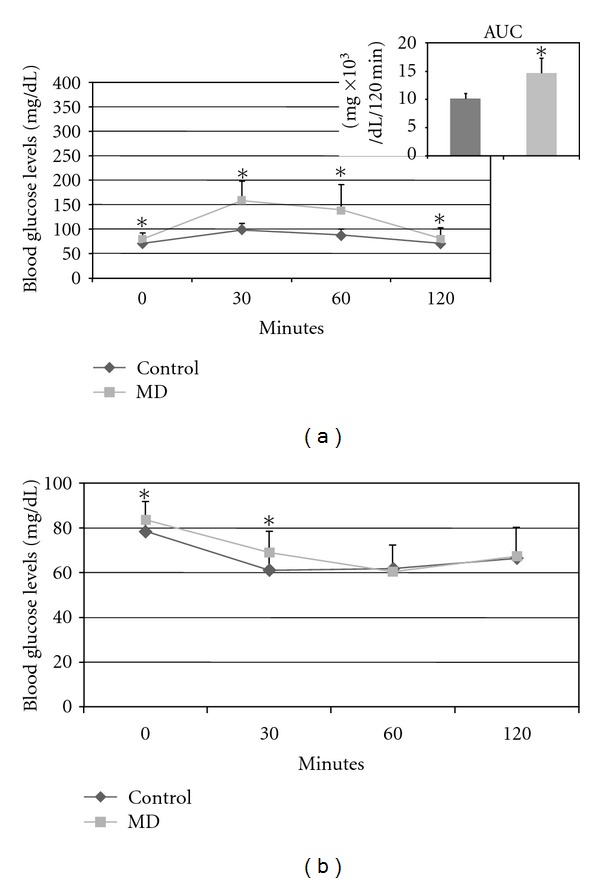
Oral glucose tolerance test (a) at day 17 of pregnancy. Area under the curve (AUC) and insulin tolerance test (b) at day 15 of pregnancy of rats with mild diabetes (MD) and nondiabetic rats (control). Data expressed as mean standard deviation.*P < 0.05—statistically significant difference compared to control group (Student *t*-test).

At days 0 and 14 of pregnancy, the blood glucose levels were higher in the diabetic rats compared to the control rats (Figure 1). Figures 2(a) and 2(b) show curves for the oral glucose tolerance test (OGTT) and insulin tolerance test (ITT), respectively. The OGTT showed an increase in blood glucose levels for diabetic rats at 0, 30, 60, and 120 min compared to control rats. Regarding the ITT test, blood glucose levels were significantly higher in MD group at two timepoints (0 and 30 min) compared to the control group. 

### 3.2. Maternal Reproductive Performance

The mean number of living fetuses, corpora lutea, and implantations was decreased, and the pre- and postimplantation loss rates were increased in MD rats compared to the control rats. The maternal weight gain of the MD rats was significantly lower compared to control group. There was a decrease in the litter weight of the MD group compared to the control group. The fetal weights in the MD group did not differ statistically from the control group (Table 1).

### 3.3. Placental Morphometry and Histopathological Analysis

**Figure 3:**
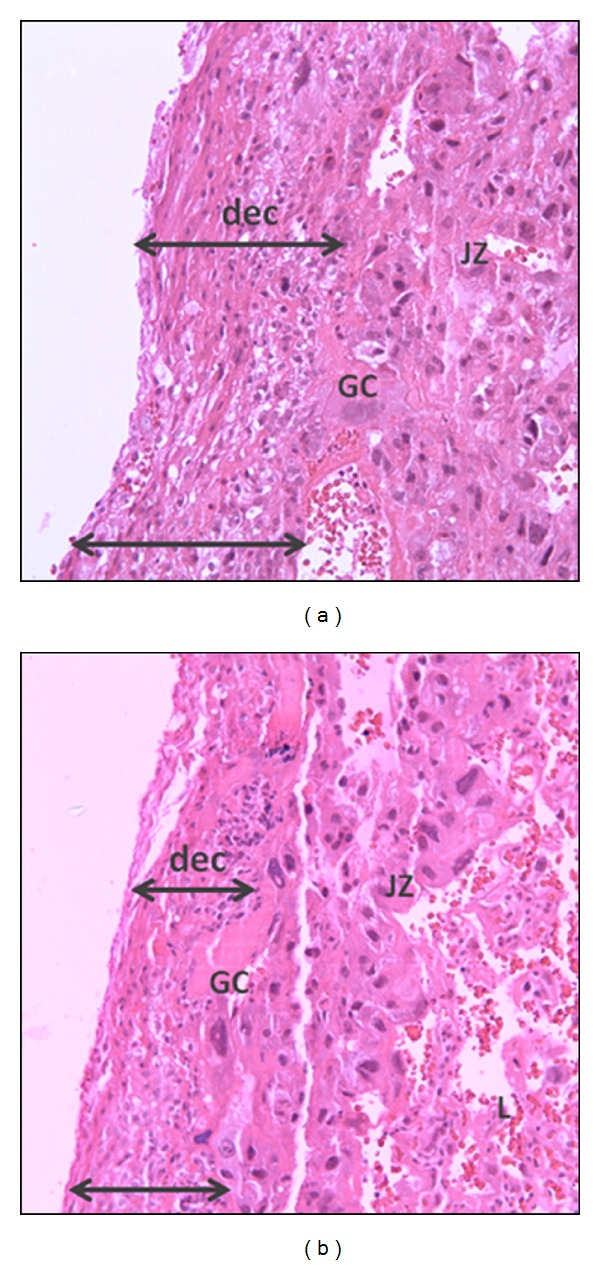
Microscopic images of the placentas (hematoxylin-eosin) at day 21 of pregnancy from nondiabetic rats (a) and mild diabetic rats (b). Arrows show the limits of decidua area. One placenta from each dam (*N* = 15) was assessed for morphometry of decidua (dec), junctional zone (JZ), and labyrinth (L). GC: giant cells. Magnification 200x.

The mean area of the decidua of the placentas in the diabetic rats was reduced compared to the control group. The mean areas of the junctional zone and labyrinth showed no significant differences between the groups (Table 2). The morphology of decidua area is represented in Figure 3. The histopathological analysis showed no changes in the placentas about inflammatory infiltrations, cystic spaces with transudate, hemorrhagic areas, and number of glycogen cells. 

## 4. Discussion

In the present study, the rats with mild diabetes showed increased glycemic means in early and middle pregnancy. Increased glycemia on day 14 of pregnancy was accompanied by glucose intolerance, shown by values altered in the oral glucose tolerance test, as well as insulin resistance, confirmed by the insulin tolerance test. These data demonstrated that this model is shown to be very similar to diabetes in pregnant women.

Exposure to hyperglycemia, under the experimental conditions in the present study, resulted in a reduced weight gain as well as a decreased litter weight in the diabetic dams when observed at the end of pregnancy. These results could be explained by the fact that the rats with mild diabetes had a smaller mean number of living fetuses, implantations, and corpora lutea as well as increased rates of embryonic losses after implantation. Although the animals of this study presented lower hyperglycemia in relation to rats with severe diabetes, they showed a reduced mean number of living fetuses and an increased rate of postembryonic implantation loss [26], thus confirming that different hyperglycemic intensities interfere with embryofetal development, as found in diabetic pregnant women [3, 4].

It is suggested that the increased glycemia prior to pregnancy or its increase in early pregnancy, as confirmed in our study, is related to the reduction in the number of corpora lutea in the diabetic animals. During oocyte development, the surrounding granulosa cells support oocyte development and provide hormonal supplementation. The hyperglycemic state influences the intercellular communication of the granulosa cells, thus altering oocyte maturation due to poor paracrine communication [27]. This would explain the reduced number of ovocyted oocytes in the mild diabetes model [28]. In many experimental diabetes models, ovulation is impaired by both ovarian- and hypothalamic-pituitary alterations [29–31]. Oocyte maturation delay alters normal zygote development [32], and this may have contributed to the increased rate of preimplantation loss and reduced number of implanted embryos in the present study. Two hypotheses can be considered to explain the enhanced percentage loss prior to embryonic implantation. The first would be the fact that the hyperglycemic insult alters the totipotent cells from zygote to a point beyond restoration, thus preventing viable embryonic development. The second would be alterations in the decidua, as seen in this study, which would delay the synchronism between decidua receptiveness and blastocyst development. Another model of mild diabetes showed a decreased uterine contractility, metabolic abnormalities during pregnancy, and a smaller number of implanted embryos [33, 34], which also corroborates our results even though diabetes induction in our experimental model occurred on the day of birth.

Our study showed a larger number of diabetic dams with embryonic deaths in relation to the control group. Maternal hyperglycemia during the early stages of development may be sufficient to program changes in physiology and metabolism which are later manifested as adverse effects in diabetic pregnancy [35]. Hence, it is remarkable that glycemic control from the beginning of pregnancy is important for placental and embryofetal development. Reproduction in women with diabetes in the preinsulin era did not differ from that of experimental animals, since miscarriages in noncontrolled human diabetes [36, 37] are equivalent to embryonic deaths (reabsorptions) in rats with severe diabetes [10, 26]. In addition, women with uncontrolled diabetes frequently presented with reproductive problems and neonatal morbidity and mortality [38–40]. The high rate of miscarriages is correlated with glucotoxicity in high concentrations, which impacts fetal viability [41]. 

There is evidence that placental alterations would be one of the factors involved in the complications for embryofetal development in diabetic pregnancies. In our mild diabetes model, the increased maternal glycemic mean observed at days 0 and 14 of pregnancy was not sufficient to cause alterations in the junctional zone or in the labyrinth area (region of maternal-fetal exchanges), as shown in the labyrinth of the placentas in rats with severe diabetes. However, it was observed that junctional area, the major endocrine area of the rat placenta, showed a structural disarrangement.

The alteration observed in decidua shows that this area is highly influenced by the hyperglycemic status, since this alteration was also observed in placentas of dams with severe diabetes (data not shown). Decidualization is a key uterine adaptation associated with the establishment of pregnancy and is characterized by the differentiation of uterine stromal cells [42]. Decidual cell differentiation is dependent on the regulatory actions of ovarian steroids, progesterone, and 17 beta-estradiol [43, 44]. Once formed, decidual cells are responsible for controlling a cascade of other changes in maternal and extraembryonic tissues necessary for pregnancy to continue establishing a protective environment, facilitating the development of the placenta and embryo [45, 46]. Garris [47] showed that diabetic pseudopregnant rats presented uterine weights and uterine blood flow depressed compared to control values, indicating uterine atrophy and poor endometrial decidualization. Besides, the progesterone levels were decreased. 

Although in our study the progesterone levels have not been measured, the reduced decidua area and reduced number of corpora lutea should be related to decreased progesterone secretion. As a result, the morphometric alteration of the decidua is directly related to the reduced number of implantations and the increased rate of embryonic losses in the group with mild diabetes observed in our study. The disorders in reproduction in diabetic rats have been associated to dysfunctions in the hypothalamic-hypophysis-gonadal axis [48], and severe diabetic dams presented reduced progesterone levels (approximately 35%) even presenting at term fetuses compared to nondiabetic dams [49]. 

## 5. Conclusion

In conclusion, mild diabetes was sufficient to trigger alterations in maternal organism leading to impaired decidua development contributing to failure in embryonic implantation and early embryonic losses. Regardless placental decidua alteration, the labyrinth, which is responsible for the maternal-fetal exchanges, showed no morphometric changes contributing to appropriate fetal development, which was able to maintain normal fetal weight at term in mild diabetic rats. Thus, this experimental model of diabetes induction at day of birth was more effective to reproduce the reproductive alterations of diabetic women.

## Figures and Tables

**Table 1:** Maternal reproductive performance of rats with mild diabetes (MD) and nondiabetic rats (control) at term pregnancy.

	Groups	
	Control (*n* = 28)	MD (*n* = 28)
Corpora lutea		
Total (*N*)	398	359
Mean SD	14.2 ± 1.6	12.4 ± 1.9*
Implantation		
Total (*N*)	374	303
Mean SD	13.4 ± 1.4	10.4 ± 2.8^∗^
Live fetuses		
Total (*N*)	359	250
Mean ± SD	12.8 ± 1.6	8.7 ± 3.4^∗^
Preimplantation loss (%)	5.7	16.1^∗^
Postimplantation loss (%)	4.0	21.3^∗^
Maternal weight gain (g)	123.1 ± 16.1	99.3 ± 25.0^∗^
Litter weight (g)	88.0 ± 10.6	62.3 ± 26.3^∗^
Fetal weight (g)	5.40 ± 0.27	5.36 ± 0.46

Data expressed as mean standard deviation.

*P < 0.05—statistically significant difference compared to control group (Student *t*-test).

**Table 2:** Mean areas of the decidua, junctional zone, and labyrinth of rats with mild diabetes (MD) and nondiabetic rats (control).

	Groups	
	Control (*n* = 15)	MD (*n* = 15)
Decidua (mm^2^)	0.087 ± 0.036	0.078 ± 0.028*
Junctional zone (mm^2^)	0.281 ± 0.061	0.297 ± 0.106
Labyrinth (mm^2^)	7.506 ± 0.997	7.725 ± 1.196

Data expressed as mean ± standard deviation.

*P < 0.05—statistically significant difference compared to control group (Student *t*-test).
